# Editorial: Reproduction-related non-coding RNAs in animals

**DOI:** 10.3389/fvets.2023.1134965

**Published:** 2023-05-03

**Authors:** Haiguang Mao, Nasser Ghanem, Sayed Haidar Abbas Raza, Hoelker Michael

**Affiliations:** ^1^School of Biological and Chemical Engineering, NingboTech University, Ningbo, China; ^2^Department of Animal Production, Faculty of Agriculture, Cairo University, Giza, Egypt; ^3^College of Animal Science and Technology, Northwest A&F University, Yangling, Shaanxi, China; ^4^Inst Anim Sci Anim Breeding and Husb, University of Bonn, Bonn, Germany

**Keywords:** reproduction, non-coding RNAs, miRNA, lncRNA, mRNA

Reproduction efficiency is an important economic trait in the production of domestic animals. In the previous several decades, it was thought that the reproduction traits were controlled by coding genes with the influence of the growing environment. Nevertheless, numerous studies have suggested that non-coding RNAs, including miRNA, lncRNA, and circRNA may play important roles in the regulation of reproduction in recent decades. With the development of transcriptomics technology, more and more non-coding RNAs related to domestic animal reproduction traits have been identified. This collection of five papers focuses on the recent insight of how non-coding RNAs affects reproduction.

Wang, Pan, et al. detected the mRNAs, miRNAs and lncRNAs in yak's primary cultured sertoli cells and leydig cells by RNA-seq. They found that the main differences between sertoli cells and leydig cells were reflected in cell proliferation and metabolism, steroid hormone synthesis and BTB dynamic regulation. The key factors caused these differences might be LAMC3, 3β-HSD, BRCA2, CNTLN, and CA2, which were regulated by non-coding RNAs.

Zhao et al. found that bta-miR-7 might play a key role in the regulation of reproduction, which might be the reason of the F1 male hybrid infertility of cattle-yak. In the early spermatocyte stage, the Usp9x-deficient spermatogonia underwent apoptosis, which subsequently leads to abnormal spermatogenesis, resulting in the complete sterility of USP9x-knockout male mice. The comprehensive regulation of these miRNAs target genes and DE genes could affect the meiosis process of spermatocyte and thus affect the reproductive traits of cattle-yak.

Tang et al. found that miRNAs could cause deformation and necrosis of ovarian tissue cells during heat stress, and ultimately disturbed the function of ovarian. Heat stress treatment was carried out by electrical heater in female rabbits. They revealed several DE miRNAs and target genes, such as miR-141-39 might target COQ6, and miR-34c-5p might potentially target Bcl-2. The negative effect of heat stress on ovaries of female rabbits was the increase of ovarian apoptosis, which might be caused by the decrease of IL-8, IL-2 in serum and the expression levels of GSH-Px and CAT, more importantly, the changes of miRNAs expression.

The roles of non-coding RNAs and their ceRNA networks of bovine follicular cyst were investigated by Wang, Cheng et al.. The result showed that lncRNA NONBTAT027373.1 could sponge miR-664b and disrupt the binding of miR-664b to HSD17B7 3'-UTR, which indicated that the lncRNAs and the target genes related to energy metabolism and steroid hormone synthesis might play key roles during the formation of cystic follicles by ceRNA mechanism.

In order to identify genes related to placenta retention and evaluate its molecular mechanism, RNA-Seq was performed on caudal vein blood samples of postpartum Holstein cows with normal discharge or retention of fetal membrane. The sequencing result demonstrated the relationship between EPAS1 and retained placenta, and confirmed that EPAS1 was upregulated in the blood of cattle experiencing retained placenta. In addition, they found that EPAS1 was negatively regulated by miR-150_R-1, and then regulated the changes of related molecules in HIF-1/ErbB signaling pathway, especially the abnormal changes of EGFR and p-FAK, which affected the adhesion of fetal placental cotyledon to uterine nodules after delivery, leading to retained placenta (1).

In conclusion, the papers gathered in this Research Topic provide an update on the impact of reproduction-related non-coding RNAs in animals.

## Author contributions

HM, NG, SR, and HM were guest associated editors of the Research Topic, they wrote and revised the editorial article. All authors contributed to the article and approved the submitted version.
